# Persistence–confidence incongruence and engagement in translation learning: a response surface analysis

**DOI:** 10.3389/fpsyg.2026.1841809

**Published:** 2026-09-07

**Authors:** Chen Chen, Zikai Guo

**Affiliations:** School of Foreign Studies, Xi'an Medical University, Xi'an, China

**Keywords:** confidence, persistence, psychological mismatch, self-efficacy, translation learning engagement, university students

## Abstract

Sustaining engagement in demanding learning contexts is a central psychological challenge, yet relatively little is known about how translation learners' internal motivational resources work together to support such engagement. The present study examined whether the alignment between persistence and confidence at baseline was prospectively associated with subsequent engagement in translation learning among Chinese university students. A two-wave survey design was employed, with 620 students participating at Wave 1 and 510 matched responses retained at Wave 2. Persistence and confidence were measured at Wave 1, and engagement in translation learning was measured 4 weeks later at Wave 2. After confirmatory factor analyses supported the distinctiveness and reliability of the focal constructs, polynomial regression with response surface analysis was conducted to test associations between congruence and incongruence in persistence and confidence and later engagement. The results showed that engagement was generally higher when persistence and confidence were aligned and increased together. However, this positive association leveled off at higher levels of congruence. Engagement was also lower when the discrepancy between persistence and confidence was larger, regardless of the direction of the mismatch. These findings suggest that engagement in translation learning is associated not only with individual psychological resources in isolation, but also with the degree to which they are internally coordinated. Because persistence and confidence were measured concurrently at Wave 1, the results should be interpreted as temporal associations rather than evidence that psychological mismatch causally reduces engagement. The study contributes to the psychology of translation learning by applying a congruence perspective to the balance between effortful persistence and capability-related confidence.

## 1 Introduction

Sustaining engagement in demanding learning contexts is fundamentally a psychological challenge. Engagement is not simply a matter of being present in class or completing assigned tasks. Rather, it reflects the extent to which learners remain behaviorally involved, cognitively invested, and affectively connected to what they are doing (Fredricks et al., 2004; Hiver et al., 2024). This challenge is especially salient in translation learning, where students must cope with ambiguity, repeated revision, and sustained cognitive effort while moving between source-text understanding and target-text production. In such a context, engagement is not a peripheral issue. It is a psychologically meaningful condition for whether learners remain actively involved in the difficult and often uncertain work of translation learning.

Recent research in translation and interpreting education has increasingly suggested that psychological processes deserve more direct attention in understanding how students learn. This psychological perspective should also be situated within the broader tradition of translation studies and translator education. Research on translator competence and pedagogy has long emphasized that translation learning involves the development of strategic, instrumental, interpersonal, and professional competences rather than linguistic transfer alone (PACTE Group, 2003; Kelly, 2005; Kiraly, 2000; European Commission, Directorate-General for Translation, 2022). From this perspective, translation learners are expected to develop not only bilingual and textual skills, but also the capacity to manage complex tasks, use resources, revise solutions, and participate in situated professional practices. The present study builds on this tradition by focusing on the psychological conditions under which students remain engaged in such demanding learning processes. Translation learning is not driven by skill alone. It also depends on how learners interpret task demands, regulate their effort, and assess their own ability to cope with difficulty. Work on L2 grit has highlighted the importance of perseverance in language-related learning (Teimouri et al., 2022), while research on translation self-efficacy has shown that learners' confidence in handling translation tasks constitutes a domain-specific psychological resource rather than a vague personal disposition (Yang et al., 2021). In related educational settings, self-efficacy has also been linked to stronger learning engagement, either directly or through broader self-regulatory processes (Doo et al., 2023; Guo et al., 2025). Within translator education more specifically, recent evidence indicates that sustained involvement in translation learning is closely tied to self-regulation and other psychology-informed learning processes (Xu and Wang Guénier, 2025; Wang and Zeng, 2026).

Even so, existing research has tended to examine persistence and confidence as separate predictors. This leaves an important question underexplored. Learners do not enter translation tasks with isolated psychological resources. They bring different internal combinations of effortful persistence and capability beliefs. A student may be highly persistent but insufficiently confident, or confident but unable to sustain effort when translation becomes difficult. These internal configurations may matter because engagement in translation learning likely depends not only on having psychological resources, but also on how well those resources work together. Related evidence from adjacent educational research suggests that discrepancy itself can shape engagement in meaningful ways (Cong et al., 2024; Song et al., 2024). Yet this configuration-based perspective remains largely absent from research on translation learning.

The present study addresses this gap by applying an established congruence perspective to the context of translation learning. Rather than claiming novelty in the response-surface framework itself, the study seeks to extend this framework to a learner-internal configuration of two translation-relevant psychological resources: persistence and confidence. Specifically, we examine whether the congruence and incongruence between persistence and confidence are associated with engagement in translation learning among Chinese university students. Rather than treating persistence and confidence as isolated linear predictors, we investigate whether engagement is better understood as the outcome of their joint psychological configuration. To do so, we adopt polynomial regression with response surface analysis, which makes it possible to test whether matched and mismatched combinations of persistence and confidence are related to different levels of engagement. In this way, the study seeks to contribute to the psychology of translation learning by shifting attention from isolated personal strengths to the internal alignment of psychological resources.

## 2 Theoretical background and hypotheses

### 2.1 Engagement in translation learning

Engagement has become a central construct in educational psychology because it captures the quality of students' active involvement in learning rather than their mere attendance or task completion. It is commonly understood as a multidimensional psychological state that includes behavioral participation, emotional involvement, and cognitive investment in academic activity (Fredricks et al., 2004; Appleton et al., 2008; Skinner et al., 2009). In higher education, engagement is also viewed as a dynamic interface between learners and their learning environments, shaped by the ways students interpret academic demands and position themselves in relation to them (Kahu, 2013; Kahu and Nelson, 2018). From this perspective, engagement is not simply a stable trait. It is a situated form of involvement that reflects how students direct their effort, attention, and affect toward learning.

A similar view has emerged in language education. Research on language learning engagement has increasingly emphasized that learners' involvement is behavioral, cognitive, affective, and often agentic, in the sense that students do not merely respond to instructional conditions but also shape them through their own contributions (Reeve and Tseng, 2011; Hiver et al., 2024). In applied linguistics, Svalberg (2009) further argued that engagement with language itself is a meaningful construct, as language learning requires not only exposure and practice but also active, reflective, and affectively charged involvement with linguistic form and meaning. This line of work is especially relevant to translation learning, where learners must constantly shift between comprehension and reformulation, monitor lexical and stylistic choices, and tolerate ambiguity across languages. Translation learning therefore places sustained demands on students' willingness to invest effort, maintain attention, and remain psychologically involved.

Although engagement has been widely studied in general education and second language learning, it has received much less focused attention in translation learning. This relative neglect is notable because translation tasks are cognitively intensive and often require repeated revision, strategic problem solving, and persistence in the face of uncertainty. Recent work in translator and interpreter education nevertheless suggests that engagement deserves more direct attention. For example, Xu and Wang Guénier (2025) showed that self-regulation was a stronger predictor of translation competence than motivation among undergraduate translation students, highlighting the importance of sustained and organized involvement in learning. In interpreter training, Yang et al. (2025) found that peer assessment enhanced not only performance but also learning engagement and self-regulated learning. More recently, Yu and Wu (2025) developed and validated an interpreting-specific engagement scale, further indicating that engagement in translation- and interpreting-related learning contexts has domain-specific features that merit dedicated investigation. Taken together, these developments suggest that engagement in translation learning is not a peripheral concern. It is a psychologically meaningful outcome through which students' motivational and self-belief resources become visible in action.

### 2.2 Persistence in translation learning

Persistence refers to the tendency to sustain effort toward long-term goals despite difficulty, frustration, or slow progress. In the broader grit literature, this tendency has typically been discussed alongside consistency of interests, but the perseverance component has often shown stronger and more reliable links with adaptive educational outcomes (Duckworth et al., 2007; Duckworth and Quinn, 2009; Credé et al., 2017). This distinction matters for the present study. Our interest is not in grit as a broad dispositional label, but in the learner's capacity to continue investing effort when translation tasks become demanding, uncertain, or mentally taxing. This emphasis is particularly relevant in language education. Building on the grit literature, Teimouri et al. (2022) conceptualized L2 grit as a domain-specific construct and demonstrated that perseverance and passion can be meaningfully examined in second language learning rather than only in general achievement settings. Their work is important because it shows that persistence in language learning is not simply a generic personality characteristic transferred into another domain. It is shaped by the specific demands of language study, including repeated practice, delayed progress, and continuous performance evaluation. In this sense, persistence may be especially consequential in translation learning, where students often encounter open-ended problems, multiple acceptable solutions, and the need to revise their work several times before reaching a satisfactory version. Recent evidence also supports a link between grit-related perseverance and engagement in language learning. Wu et al. (2024), for instance, reported that grit was positively associated with second language learning engagement, with affective balance helping to explain part of this relationship. Such findings are relevant here because translation learning may be even more effortful than many ordinary classroom language tasks. Learners must remain engaged not only while understanding source-language meaning but also while generating and evaluating target-language alternatives. In practice, students who persist are more likely to keep working through ambiguity, tolerate imperfect intermediate drafts, and remain engaged when immediate success is not guaranteed. For this reason, the present study focuses specifically on persistence, understood as sustained effort and follow-through, as one of the two core psychological resources that may shape engagement in translation learning.

### 2.3 Confidence in translation learning

Confidence in the present study is grounded in the concept of self-efficacy, namely, learners' beliefs about their capability to perform tasks successfully under given conditions. In social cognitive theory, self-efficacy is not a vague sense of optimism. It refers to a person's judgment of what they can do with the skills they possess, and it is consequential because such judgments shape effort, persistence, emotional reactions, and resilience in the face of challenge (Bandura, 1993). Self-efficacy beliefs develop through multiple sources, including mastery experience, vicarious experience, social persuasion, and interpretations of physiological or affective states (Usher and Pajares, 2008). In educational settings, these beliefs are closely tied to how students approach difficult tasks, how long they persist, and how they interpret temporary setbacks. A substantial body of research has shown that academic self-efficacy is associated with adaptive learning processes and achievement. In higher education, self-efficacy has been linked to performance directly and also indirectly through motivational and self-regulatory mechanisms (Honicke and Broadbent, 2016). Students who believe they can manage academic demands are generally more willing to invest in those demands, especially when success requires planning, monitoring, and sustained effort. At the same time, self-efficacy is domain-sensitive. The confidence that matters for mathematics, writing, or public speaking is not identical across contexts, because learners interpret task demands through domain-specific experience.

This point is particularly important in translator education. Translation self-efficacy concerns learners' beliefs about their ability to understand source texts, solve translation problems, choose appropriate strategies, and produce adequate target-language renditions under given task conditions. Existing research has begun to clarify both the meaning and the educational relevance of this construct. Haro-Soler and Kiraly (2019) showed that pedagogical practices and collaborative classroom arrangements can shape translation students' self-efficacy beliefs. Yang et al. (2021) further provided psychometric evidence for a translation self-efficacy scale developed for Chinese learners of translation, offering a more domain-specific basis for studying confidence in translation learning. More recently, Abdel Latif and Alhamad (2025) reviewed published measures of translation and interpreting self-efficacy and concluded that this area remains underdeveloped but theoretically significant, particularly because self-efficacy is likely to matter for both performance and perseverance in translator and interpreter training. Empirical findings also suggest that translation self-efficacy is closely related to actual learning processes and outcomes. For example, Lu et al. (2022) found that translation self-efficacy mediated the relationship between online information seeking and translation performance among translation students. This is important because it implies that confidence is not merely an affective by-product of doing well. It can also function as a psychological mechanism through which learning behaviors are translated into better task performance. In the present study, confidence is therefore conceptualized as learners' perceived capability to cope effectively with translation-related demands. It is treated as a second key psychological resource that may shape how deeply students engage in translation learning.

### 2.4 Psychological mismatch between persistence and confidence

Although persistence and confidence are each meaningful in their own right, there are reasons to expect that their joint configuration may matter more than either resource considered in isolation. Engagement in translation learning requires not only the willingness to keep going but also the belief that one can manage the task at hand. A learner who is prepared to persist but doubts their capability may remain in the task under tension, investing effort while experiencing hesitation, insecurity, or psychological strain. By contrast, a learner who feels capable but lacks persistence may approach translation tasks with confidence at the outset yet fail to maintain effort when difficulty accumulates. In both cases, one psychological resource is available while the other is insufficient, creating a form of internal imbalance that may undermine stable engagement. This logic is consistent with broader perspectives on engagement. Educational research has repeatedly shown that engagement is shaped by how students interpret demands, evaluate their competence, and regulate their participation over time (Skinner et al., 2009; Kahu, 2013). From a self-efficacy perspective, confidence should matter because it affects how learners respond to challenge (Bandura, 1993). From a grit or perseverance perspective, persistence should matter because it sustains effort across extended difficulty (Duckworth et al., 2007; Teimouri et al., 2022). Yet in demanding learning settings such as translation, these two resources are unlikely to operate independently. What seems especially important is whether they can work together in a coordinated way. Sustained engagement may therefore require not simply high persistence or high confidence, but a workable alignment between the willingness to persist and the belief that one can cope effectively.

The focus on persistence and confidence is also consistent with broader motivational theories in educational psychology. Achievement emotion research suggests that learners' appraisals of control and value shape their emotional and behavioral involvement in learning (Pekrun, 2006). Work on implicit theories and achievement goals further indicates that students' beliefs about ability and their reasons for engaging in achievement tasks can influence effort, challenge-seeking, and persistence (Dweck, 2006; Elliot, 1999). These perspectives converge on the idea that engagement is shaped by a coordinated motivational system rather than by a single isolated attribute.

Recent work in translation and interpreting education supports this general expectation, even though the question has rarely been framed explicitly in terms of congruence or mismatch. Xu and Wang Guénier (2025) showed that motivation alone was not enough to sustain strong translation competence when self-regulation was weak, suggesting that isolated psychological resources may not be sufficient in the absence of complementary processes. In interpreter training, Yang et al. (2025) found that peer assessment enhanced engagement together with confidence, reflection, and strategic learning. Meanwhile, Wang (2025) reported that confidence-related anxiety remained a salient issue in interpreting learning, even when other aspects of anxiety were alleviated. Taken together, these studies suggest that successful engagement in translation- and interpreting-related learning depends on a coordinated psychological system rather than on a single motivational factor. A mismatch perspective is also supported by educational research beyond translation learning. Using response surface analysis, Song et al. (2024) showed that congruence and discrepancy between adolescents' and parents' educational expectations were differentially related to study engagement. The present study extends this logic from an interindividual discrepancy to an intraindividual discrepancy. In interindividual congruence research, the focal question is whether two people or social agents hold aligned expectations or perceptions. In the present study, the focal question is whether two psychological resources within the same learner are aligned. These two forms of discrepancy are not identical, but they share a common configuration-based premise: educational outcomes may depend not only on the absolute level of a given resource, but also on the degree of correspondence between theoretically related components. Therefore, the prior congruence literature provides a methodological and conceptual basis for examining whether learner-internal alignment between persistence and confidence is associated with engagement in translation learning. Although their variables differ from those in the present study, the broader implication is important: discrepancy itself can have meaningful consequences for educational engagement, and these consequences may be obscured when researchers focus only on separate main effects. Applying this logic to translation learning suggests that the cost of psychological mismatch may lie precisely in the learner's internal configuration. When persistence and confidence diverge, engagement may weaken not because either resource is irrelevant, but because the learner's motivational system lacks coherence.

### 2.5 The present study and hypotheses

The foregoing review suggests three points. First, engagement in translation learning is a psychologically meaningful outcome because it captures students' active behavioral, cognitive, and emotional involvement in demanding translation tasks. Second, persistence and confidence each appear to be relevant resources for such involvement. Third, existing research has tended to examine these resources separately, while paying much less attention to whether their alignment or divergence shapes engagement in translation learning. This gap is particularly notable in translator education, where students must often sustain effort under uncertainty while also relying on beliefs about their capability to solve translation problems. The present study addresses this gap by examining the joint role of persistence and confidence in Chinese university students engaged in translation learning. Rather than treating the two variables as isolated predictors, we investigate whether their congruence and incongruence are associated with different levels of engagement. To do so, we adopt polynomial regression with response surface analysis, which allows us to move beyond simple additive relations and evaluate whether matched and mismatched configurations of persistence and confidence are differentially related to engagement.

Based on the literature reviewed above, two hypotheses were advanced.

**H1**. When persistence and confidence are congruent, engagement in translation learning will be higher when both are high than when both are low.
**H2**. Larger discrepancy between persistence and confidence will be associated with lower engagement in translation learning.

## 3 Materials and methods

### 3.1 Participants and procedure

This study employed a two-wave questionnaire design with a 4-week interval. The timing was anchored to one complete translation-assignment cycle in the participating courses. Wave 1 was administered at the beginning of the cycle, when the translation task was introduced. During Week 1, students analyzed the source text and planned their translation approach; during Week 2, they prepared an initial translation and discussed relevant translation strategies; during Week 3, they received instructor and peer feedback; and during Week 4, they revised and submitted the translation. Wave 2 was administered after completion of the revision and submission stage. The interval therefore corresponded to a concrete curricular unit rather than an arbitrary period of temporal separation. A 4-week window is also consistent with prior longitudinal engagement research. In an EFL context, Alzahrani (2023) repeatedly assessed undergraduate students' classroom engagement across a 4-week instructional period and identified meaningful variation in cognitive and social engagement across the weekly sessions. In higher education, Ganotice et al. (2024) measured students' motivation at baseline and assessed their course-related engagement and disaffection 4 weeks later following a 4-week curricular program. Although these studies do not establish 4 weeks as a universally optimal interval, they show that engagement can be meaningfully assessed over a bounded 4-week instructional period and that baseline motivational resources can be prospectively related to engagement measured at the end of that period. In the present study, the interval served two purposes: it reduced same-occasion reporting between the psychological predictors and the outcome, and it allowed engagement to be assessed after students had completed a full cycle of translation practice, feedback, and revision. Because engagement was measured only at Wave 2, however, the design was not intended to estimate within-person change in engagement from baseline. Nor can it determine whether the association between the baseline persistence–confidence configuration and engagement remains stable, strengthens, or decays over longer intervals. Addressing those questions would require repeated measurements of both the psychological configuration and engagement across multiple instructional cycles. The participants were Chinese university students engaged in translation learning.

To be eligible for inclusion, students had to be currently enrolled in at least one university course with a substantial translation component and to have received formal translation instruction for no less than one semester. At Wave 1, 620 students completed the baseline survey. Four weeks later, the follow-up survey was distributed to the same cohort. The two waves were matched through anonymous self-generated identification codes. After screening the data for duplicate submissions, straight-lining across all substantive items, and unsuccessful matching across the two waves, 510 valid cases were retained for the final analysis, corresponding to a retention rate of 82.3%.

Participation was voluntary. Before entering the questionnaire, all respondents read an information sheet explaining the purpose of the study, the anonymous nature of the survey, and their right to discontinue participation at any point. No personally identifying information was collected. To reduce common method inflation, the focal predictors were measured at Wave 1, whereas the outcome variable was measured at Wave 2. The baseline survey also collected background information, including gender, year of study, major type, self-rated second-language proficiency, length of translation-learning experience, and weekly frequency of translation practice. The questionnaire was administered in Chinese. The competence-based translation self-efficacy scale was used in its original Chinese form. The adapted persistence and engagement items were translated and back-translated following the procedure proposed by Brislin (1970). Minor wording adjustments were introduced only to anchor the items in the context of translation learning while preserving their original meaning. Because the online survey platform required a response before submission, the matched dataset contained no item-level missing values.

### 3.2 Measures

All substantive variables were assessed with published instruments. Each scale used five response categories, and higher scores consistently indicated higher levels of the target construct. Composite scores were computed by averaging the relevant item responses.

#### 3.2.1 Persistence

Persistence was operationalized as the perseverance-of-effort component of L2 grit. It was measured with the five perseverance items from the domain-specific L2 grit scale developed by Teimouri et al. (2022). In the present study, the wording of the items was minimally adapted so that they referred to translation learning rather than second-language learning in general. The adapted items captured diligence, sustained effort, and resistance to setbacks in translation learning. Responses ranged from 1 (*not like me at all*) to 5 (*very much like me*). This operationalization was chosen because the title and theoretical model of the study focus on persistence rather than on the broader two-dimensional construct of grit. Using the perseverance subscale therefore provided a closer match between the theoretical argument and the empirical measure.

#### 3.2.2 Confidence

Confidence in translation learning was measured with the competence-centered part of the Translating Self-Efficacy scale for Chinese learners of translation developed by Yang et al. (2021). Specifically, we used the 12 items reflecting efficacy in internal competence and efficacy in external competence. These items assess students' beliefs about their capability to understand source texts, identify translation problems, select appropriate strategies, use digital resources effectively, and respond to translation demands in a competent manner. Responses were recorded on a five-point scale ranging from 1 (*strongly disagree*) to 5 (*strongly agree*).

The decision to use the 12 competence-centered items was made based on theoretical considerations before conducting the main polynomial regression and response-surface analyses. In the present study, confidence was conceptualized as capability-related self-efficacy, referring to learners' perceived ability to perform translation-related tasks successfully. By contrast, persistence was conceptualized as sustained effort and follow-through when facing difficulty. The three psycho-physical items in the original TSE-C were not included because they primarily reflect effort regulation, endurance, and psychological reactions during task engagement, which overlap conceptually with persistence and may weaken the distinction between the two focal constructs. Therefore, excluding these items was intended to establish a clearer construct boundary rather than to maximize statistical associations. To further evaluate this measurement decision, we conducted additional confirmatory factor analyses comparing the full 15-item TSE-C specification with the reduced 12-item competence-centered specification.

#### 3.2.3 Engagement in translation learning

Engagement in translation learning was assessed at Wave 2 with the University Student Engagement Inventory, originally developed by Maroco et al. (2016) and later validated in Chinese university samples by She et al. (2023). The USEI conceptualizes engagement as a multidimensional construct comprising behavioral, emotional, and cognitive engagement. We retained the original 15 items and introduced only minimal contextual adaptations so that the items referred specifically to translation learning rather than to university study in general. The behavioral items assessed students' effortful participation and task completion in translation learning. The emotional items captured enthusiasm, interest, and affective involvement in translation coursework. The cognitive items focused on reflective, self-regulatory, and meaning-oriented learning processes. Responses ranged from 1 (*never*) to 5 (*always*). Following the original logic of the instrument, engagement was treated as a higher-order construct reflected in its behavioral, emotional, and cognitive components. A global engagement score was obtained by averaging all 15 items.

#### 3.2.4 Control variables

Six control variables were included in the analyses: gender, year of study, major type, self-rated second-language proficiency, length of translation-learning experience, and weekly frequency of translation practice. These variables were entered as controls because they may plausibly relate to students' translation-learning engagement and could therefore confound the association between the focal predictors and the outcome.

### 3.3 Analytic strategy

Data analysis proceeded in two stages. First, the psychometric properties of the study measures were examined. Confirmatory factor analyses were conducted in lavaan (Rosseel, 2012) to test the distinctiveness of persistence, confidence, and engagement. Given the ordinal nature of the items, the CFA models were estimated with the weighted least squares mean and variance adjusted estimator. Because the distinction between persistence and confidence was theoretically crucial, the hypothesized measurement structure was compared with more parsimonious alternatives in which the two constructs were merged. Internal consistency was evaluated with Cronbach's alpha and McDonald's omega. Following conventional practice, model fit was judged by the comparative fit index, Tucker–Lewis index, root mean square error of approximation, and standardized root mean square residual (Hu and Bentler, 1999).

Second, the substantive hypotheses were tested through polynomial regression with response surface analysis (Edwards and Parry, 1993; Shanock et al., 2010; Humberg et al., 2019). This method was selected because the present study does not merely concern the independent effects of persistence and confidence. Rather, it is concerned with whether their alignment and divergence are associated with different levels of engagement in translation learning. Difference scores are poorly suited to address such questions, whereas polynomial regression allows the joint pattern of the two predictors to be modeled directly. The WLSMV estimator was used only for the item-level confirmatory factor analyses. After composite scores were computed by averaging the relevant scale items, the polynomial regression and response-surface analyses were estimated using ordinary least squares (OLS). Accordingly, the standard errors reported for the polynomial regression coefficients and response-surface parameters are OLS-based rather than WLSMV-based. Before estimation, persistence and confidence were mean-centered. We then computed the quadratic and interaction terms required for response surface analysis. Engagement in translation learning at Wave 2 was regressed on persistence, confidence, their higher-order terms, and the set of controls:

Because engagement was modeled as a higher-order construct in the measurement model, the main polynomial regression used the global engagement composite as the outcome variable. This decision was based on the theoretical premise that behavioral, emotional, and cognitive engagement jointly reflect students' overall involvement in translation learning, rather than three unrelated outcomes. The higher-order CFA also supported this dimensional structure. Therefore, the present analysis prioritized the overall engagement construct to maintain consistency between the measurement model and the substantive hypotheses. We acknowledge, however, that the persistence–confidence configuration may operate differently across behavioral, emotional, and cognitive engagement, and future research should examine these dimension-specific patterns more directly. The polynomial regression model and the four derived response-surface parameters are presented in Equations 1–5.


$$\begin{array}{l} E=b_{0}+b_{1}P+b_{2}C+b_{3}P^{2}+b_{4}PC+b_{5}C^{2}+\overset{m}{\underset{k=1}{\sum }}\gamma_{k}X_{k}+\varepsilon , \end{array}$$ (1)

where *E* denotes engagement in translation learning, *P* denotes persistence, *C* denotes confidence, and *X*_*k*_ denotes the control variables.

The fitted response surface was interpreted through the conventional surface parameters. The slope along the line of congruence, where persistence equals confidence, was calculated as:


$$\begin{array}{l} a_{1}=b_{1}+b_{2}, \end{array}$$ (2)

and the curvature along the same line was calculated as:


$$\begin{array}{l} a_{2}=b_{3}+b_{4}+b_{5}. \end{array}$$ (3)

The slope along the line of incongruence, where persistence and confidence diverge, was calculated as:


$$\begin{array}{l} a_{3}=b_{1}-b_{2}, \end{array}$$ (4)

and the curvature along this line was calculated as:


$$\begin{array}{l} a_{4}=b_{3}-b_{4}+b_{5}. \end{array}$$ (5)

Our mismatch hypothesis would be supported if the curvature along the incongruence line was significantly negative, indicating that engagement declines as the discrepancy between persistence and confidence increases. In addition to the numerical tests, three-dimensional response surface plots were inspected to visualize the joint pattern of the two predictors. All statistical tests were two-tailed, with the significance level set at 0.05.

## 4 Statistical analysis results

### 4.1 Sample characteristics

Of the 620 students who completed the baseline survey at Wave 1, 510 provided matched and valid responses at Wave 2 and were retained for the final analyses, yielding a retention rate of 82.3%. The characteristics of the matched sample are presented in Table 1. Female students constituted the majority of the sample (73.7%), whereas male students accounted for 26.3%. In terms of year of study, second-year students formed the largest group (30.8%), followed by third-year students (27.6%), first-year students (24.1%), and fourth-year or above students (17.5%). The sample also reflected a relatively diverse academic background. Students majoring in English-related fields represented the largest subgroup (34.7%), followed by those in translation-related majors (29.2%), other majors (22.2%), and other language-related majors (13.9%). With respect to self-rated second-language proficiency, most participants identified themselves as being at the intermediate level (38.6%) or lower-intermediate level (24.1%). Smaller proportions reported upper-intermediate proficiency (22.0%), high proficiency (8.2%), or low proficiency (7.1%). Regarding prior experience in translation learning, the largest proportion of students reported 1–2 years of experience (35.3%), followed by 2–3 years (25.3%), more than 3 years (21.0%), and less than 1 year (18.4%). Weekly translation practice also varied across the sample. Most participants reported engaging in translation practice for 1–3 h per week (42.2%), while 27.1% reported four to 6 h, 18.0% reported more than 6 h, and 12.7% reported less than 1 h. Overall, these characteristics suggest that the matched sample captured a reasonably broad range of university learners situated in translation-learning contexts.

**Table 1:** Sample characteristics of the matched participants.

Characteristic	Category	*n*	%
Gender	Female	376	73.7
	Male	134	26.3
Year of study	First year	123	24.1
	Second year	157	30.8
	Third year	141	27.6
	Fourth year or above	89	17.5
Major type	Translation-related major	149	29.2
	English-related major	177	34.7
	Other language-related major	71	13.9
	Other major	113	22.2
Self-rated L2 proficiency	Low	36	7.1
	Lower-intermediate	123	24.1
	Intermediate	197	38.6
	Upper-intermediate	112	22.0
	High	42	8.2
Length of translation-learning experience	Less than 1 year	94	18.4
	1 to 2 years	180	35.3
	2 to 3 years	129	25.3
	More than 3 years	107	21.0
Weekly translation practice	Less than 1 h	65	12.7
	1 to 3 h	215	42.2
	4 to 6 h	138	27.1
	More than 6 h	92	18.0

We also conducted an attrition analysis to examine whether the 510 retained participants differed from the 110 students who completed only the Wave 1 survey. Chi-square tests were used for categorical background variables, and independent-samples *t* tests were used for continuous Wave 1 variables. The retained and dropped participants did not differ significantly in gender [χ^2^(1) = 0.45, *p* = 0.502], year of study [χ^2^_(3)_ = 2.18, *p* = 0.536], major type [χ^2^_(3)_ = 1.34, *p* = 0.720], self-rated second-language proficiency [χ^2^_(4)_ = 3.21, *p* = 0.523], length of translation-learning experience [χ^2^_(3)_ = 0.98, *p* = 0.806], weekly translation practice [χ^2^_(3)_ = 1.87, *p* = 0.599], Wave 1 persistence [*t*_(608)_ = 0.84, *p* = 0.401], or Wave 1 confidence [*t*_(608)_ = –0.52, *p* = 0.603]. These results suggest that attrition was not systematically associated with the available baseline demographic or psychological variables.

### 4.2 Measurement model, reliability, and discriminant validity

We first examined the adequacy of the measurement model before conducting the substantive analyses. The hypothesized model, which specified persistence, confidence, and a second-order engagement construct reflected by behavioral, emotional, and cognitive engagement, showed an excellent fit to the data, χ^2^_(458)_ = 504.43, CFI = 0.998, TLI = 0.997, RMSEA = 0.014, and SRMR = 0.028, as shown in Table 2. These results indicate that the proposed measurement structure provided a highly satisfactory representation of the observed data. To further evaluate discriminant validity, the hypothesized model was compared with several alternative models. When persistence and confidence were combined into a single factor while engagement was retained as a separate construct, model fit became weaker, χ^2^_(460)_ = 852.47, CFI = 0.980, TLI = 0.979, RMSEA = 0.041, and SRMR = 0.042. Likewise, the model in which behavioral, emotional, and cognitive engagement were collapsed into a single first-order factor also fit the data less well, χ^2^_(461)_ = 775.71, CFI = 0.984, TLI = 0.983, RMSEA = 0.037, and SRMR = 0.036. The single-factor model showed a particularly poor fit, χ^2^_(464)_ = 4,977.45, CFI = 0.772, TLI = 0.756, RMSEA = 0.138, and SRMR = 0.149. Taken together, these comparisons support the distinctiveness of persistence, confidence, and engagement, while also justifying the treatment of engagement as a higher-order construct.

**Table 2:** Fit indices for the competing measurement models.

Model	χ^2^	*df*	CFI	TLI	RMSEA	SRMR
Hypothesized model: persistence, confidence, and higher-order engagement	504.43	458	0.998	0.997	0.014	0.028
Persistence and confidence combined; engagement separate	852.47	460	0.980	0.979	0.041	0.042
Persistence separate; confidence separate; engagement collapsed into one first-order factor	775.71	461	0.984	0.983	0.037	0.036
Single-factor model	4,977.45	464	0.772	0.756	0.138	0.149

CFI, comparative fit index; TLI, Tucker Lewis index; RMSEA, root mean square error of approximation; SRMR, standardized root mean square residual.

Because all focal variables were assessed through self-report questionnaires, we also considered the possibility of common-method bias. The poor fit of the single-factor model provides evidence against the interpretation that the observed associations were mainly attributable to a single common-method factor. Although this test cannot completely rule out common-method influence, it suggests that common-method variance was unlikely to fully account for the distinctiveness of persistence, confidence, and engagement in the present data.

Because the distinction between persistence and confidence was central to the present study, we further examined whether the competence-centered 12-item confidence measure provided an empirically adequate representation compared with the full 15-item TSE-C specification. Confirmatory factor analyses were conducted by comparing the full and reduced confidence specifications within the complete measurement model. As shown in Table 3, both models demonstrated excellent fit to the data, and the reduced 12-item specification showed slightly better global fit indices than the full 15-item specification (CFI = 0.998 vs. 0.995; TLI = 0.997 vs. 0.994; RMSEA = 0.014 vs. 0.016; SRMR = 0.028 vs. 0.033). However, because global fit indices are sensitive to model complexity and the number of indicators, this comparison alone cannot establish that the retained confidence indicators are distinct from the persistence indicators. We therefore supplemented the global model comparison with item-level factor loadings, AVE-based comparisons, an HTMT assessment, and a diagnostic analysis of the three excluded psycho-physical items.

**Table 3:** CFA model comparison between the full and reduced confidence specifications.

Measurement model	χ^2^	*df*	CFI	TLI	RMSEA	SRMR
Full model (15-item confidence)	621.37	552	0.995	0.994	0.016	0.033
Reduced model (12-item confidence)	504.43	458	0.998	0.997	0.014	0.028

To examine the separation of the retained indicators directly, Table 4 reports the standardized item-level factor loadings for persistence and confidence within the complete measurement model. The five persistence items loaded strongly on their intended factor, with standardized loadings ranging from 0.774 to 0.825, and the 12 retained confidence items likewise showed substantial loadings on their intended factor, ranging from 0.743 to 0.847. All item-level loadings were statistically significant at *p* < 0.001. Formal discriminant-validity tests provided convergent evidence that persistence and confidence were empirically distinguishable. The square roots of the AVE values for persistence and confidence were 0.800 and 0.792, respectively, and both exceeded the CFA latent correlation between the two constructs (ϕ = 0.562). The AVE values for persistence (0.640) and confidence (0.628) also exceeded their shared latent variance (ϕ^2^ = 0.316). In addition, the HTMT ratio between persistence and confidence was 0.548, which was below the conservative criterion of 0.850. Collectively, these results support the discriminant validity of the retained persistence and competence-centered confidence measures.

**Table 4:** Item-level factor loadings and discriminant validity evidence for persistence and confidence.

Construct and item	Std.all (λ)	SE	*z*	*p*
Panel A. Standardized factor loadings from the full measurement model				
Persistence (*P*)				
P1: Sustaining effort in translation	0.774	0.028	27.64	< 0.001
P2: Overcoming translation difficulty	0.816	0.026	31.38	< 0.001
P3: Diligence in continuous practice	0.825	0.026	31.73	< 0.001
P4: Resistance to task setbacks	0.783	0.027	29.00	< 0.001
P5: Task completion despite fatigue	0.801	0.027	29.67	< 0.001
Confidence (*C*)				
C1: Understanding source text nuances	0.762	0.027	28.22	< 0.001
C2: Identifying translation problems	0.785	0.026	30.19	< 0.001
C3: Selecting translation strategies	0.814	0.024	33.92	< 0.001
C4: Utilizing digital resources	0.743	0.028	26.54	< 0.001
C5: Ensuring terminology accuracy	0.821	0.024	34.21	< 0.001
C6: Evaluating target text quality	0.847	0.023	36.83	< 0.001
C7: Adapting style to domain demands	0.776	0.026	29.85	< 0.001
C8: Proofreading and self-correction	0.793	0.025	31.72	< 0.001
C9: Resolving semantic ambiguities	0.808	0.025	32.32	< 0.001
C10: Managing translation workflow	0.751	0.027	27.81	< 0.001
C11: Producing fluent renditions	0.832	0.023	36.17	< 0.001
C12: Meeting quality standards	0.771	0.026	29.65	< 0.001
Panel B. Discriminant validity parameters				
Persistence AVE = 0.640 ($\sqrt{\text{AVE}}=0.800$)			HTMT ratio = 0.548	
Confidence AVE = 0.628 ($\sqrt{\text{AVE}}=0.792$)			CFA latent correlation ϕ = 0.562	
Shared latent variance ϕ^2^ = 0.316			HTMT criterion: < 0.850	
Panel C. Diagnostic loadings of excluded psycho-physical items (15-item auxiliary model)				
*Item code and content*	*Primary loading on *C**	*Cross-loading on *P**	*Cross-loading SE*	*Cross-loading *p**
TSE_PP1: Physical stamina	0.528 (*p* < 0.001)	0.443	0.048	< 0.001
TSE_PP2: Mental focus	0.493 (*p* < 0.001)	0.471	0.046	< 0.001
TSE_PP3: Translation fatigue	0.536 (*p* < 0.001)	0.418	0.049	< 0.001

All standardized factor loadings (Std.all) were estimated using WLSMV. The estimates in Panel **(A)** were obtained from the complete measurement model comprising Persistence, Confidence, and the higher-order Engagement construct reflected by behavioral, emotional, and cognitive engagement, χ^2^ (458) = 504.43, CFI = 0.998. The CFA latent correlation, ϕ = 0.562, is the standardized correlation between the latent Persistence and Confidence factors and is distinct from the composite-score Pearson correlation (*r* = 0.510). The square root of the AVE for each construct exceeded the latent correlation, each AVE exceeded the shared latent variance, and the HTMT ratio was below the criterion of 0.850, collectively supporting discriminant validity. In Panel **(C)**, the three excluded psycho-physical items were allowed to load simultaneously on Confidence and Persistence in the auxiliary 15-item Confidence model; the reported primary and cross-loadings were therefore obtained from the same model. Item descriptions are abbreviated for presentation.

To examine the potential overlap introduced by the three excluded psycho-physical items, we also estimated an auxiliary model using the full 15-item confidence specification and allowed these three items to load simultaneously on confidence and persistence. As shown in Panel C of Table 4, their primary loadings on confidence ranged from 0.493 to 0.536, whereas their cross-loadings on persistence ranged from 0.418 to 0.471. All primary and cross-loadings were statistically significant at *p* < 0.001. These findings indicate that the psycho-physical items retained meaningful associations with confidence but also captured substantial persistence-related variance. Their exclusion therefore helped preserve the intended distinction between capability-related confidence and sustained effort without compromising the reliability or convergent validity of the retained confidence measure.

The reliability and convergent validity results are reported in Table 5. For persistence, the standardized factor loadings ranged from 0.77 to 0.83, Cronbach's alpha was 0.877, McDonald's omega was 0.877, and the average variance extracted was 0.640. Confidence likewise demonstrated strong psychometric properties, with factor loadings ranging from 0.74 to 0.85, alpha = 0.942, omega = 0.942, and AVE = 0.628. The three first-order engagement dimensions also showed satisfactory measurement quality. Behavioral engagement yielded loadings from 0.79 to 0.85, alpha = 0.895, omega = 0.895, and AVE = 0.684. Emotional engagement showed loadings from 0.76 to 0.88, alpha = 0.888, omega = 0.888, and AVE = 0.664. Cognitive engagement showed loadings from 0.79 to 0.86, alpha = 0.899, omega = 0.899, and AVE = 0.693. At the higher-order level, overall engagement was strongly reflected by its three first-order dimensions, with standardized higher-order loadings ranging from 0.91 to 0.92. The higher-order engagement construct also showed excellent internal consistency, with alpha = 0.949 and omega = 0.949, and strong convergent validity, with an AVE of 0.840. Overall, these findings indicate that all focal constructs possessed satisfactory reliability and convergent validity, and that the measurement model was adequate for the subsequent polynomial regression and response-surface analyses.

**Table 5:** Standardized loadings, reliability, and convergent validity of the study measures.

Construct	Number of items	Loading range	α	ω	AVE
Persistence	5	0.77–0.83	0.877	0.877	0.640
Confidence	12	0.74–0.85	0.942	0.942	0.628
Behavioral engagement	5	0.79–0.85	0.895	0.895	0.684
Emotional engagement	5	0.76–0.88	0.888	0.888	0.664
Cognitive engagement	5	0.79–0.86	0.899	0.899	0.693
Overall engagement	15	Higher-order: 0.91–0.92	0.949	0.949	0.840

AVE, average variance extracted. For overall engagement, the loading range refers to the standardized higher-order loadings from the second-order engagement factor to the three first-order engagement dimensions.

### 4.3 Descriptive statistics and zero-order correlations

Table 6 presents the descriptive statistics and zero-order correlations for the focal variables. The mean score for persistence was 3.12 (*SD* = 0.97), the mean score for confidence was 3.19 (*SD* = 0.95), and the mean score for overall engagement in translation learning was 3.20 (*SD* = 0.98). These values suggest that, on average, the participants reported moderate levels of persistence, confidence, and engagement. Persistence and confidence were positively correlated (*r* = 0.51), indicating that students who reported stronger persistence in translation learning also tended to report greater confidence in their translation-related capability beliefs. This association was moderate in magnitude and remained consistent with the distinctiveness of the two constructs established in the confirmatory factor analyses. In addition, persistence was positively associated with overall engagement (*r* = 0.47), and confidence was likewise positively associated with overall engagement (*r* = 0.46). These correlations suggest that both psychological resources were linked to greater involvement in translation learning at the bivariate level. Overall, the zero-order correlations were in the expected direction and provided an initial basis for the subsequent polynomial regression and response-surface analyses. At the same time, the moderate overlap between persistence and confidence further supports the need to examine not only their independent associations with engagement but also the potential consequences of their alignment and divergence.

**Table 6:** Descriptive statistics and zero-order correlations among the focal variables.

Variable	*M*	*SD*	1	2	3
1. Persistence	3.12	0.97	–		
2. Confidence	3.19	0.95	0.51	–	
3. Overall engagement	3.20	0.98	0.47	0.46	–

All coefficients are Pearson correlations. Values on the diagonal are omitted.

### 4.4 Polynomial regression results for engagement in translation learning

We next examined whether persistence and confidence jointly predicted engagement in translation learning beyond the effects of the control variables. As shown in Table 7, Model 1 included only the control variables and explained a relatively small proportion of the variance in engagement (*R*^2^ = 0.017). This control-only model was not statistically significant (*F* = 0.99, *p* = 0.443), indicating that the included background variables had limited explanatory power when considered alone. The controls were nevertheless retained because they had been selected *a priori* as plausible background covariates and allowed the focal persistence–confidence associations to be interpreted after adjusting for basic demographic, proficiency, experience, and practice-related differences. Adding the linear terms of persistence and confidence in Model 2 increased the explained variance to 0.160, yielding an increment of 0.143. In this model, persistence showed a significant positive association with engagement (*b* = 0.311, *p* < 0.001), whereas the linear effect of confidence did not reach statistical significance (*b* = 0.115, *p*>0.05). This pattern suggests that persistence was the more robust linear correlate of engagement at this stage of the analysis.

**Table 7:** Polynomial regression predicting engagement in translation learning.

Predictor	Model 1	Model 2	Model 3
Control variables			
Gender: Male (ref = Female)	–0.072	–0.052	–0.063
Year of study	0.001	–0.014	–0.016
Major: English-related (ref = Translation-related)	0.116	0.140	0.151
Major: Other language-related (ref = Translation-related)	0.206	0.130	0.142
Major: Other major (ref = Translation-related)	0.163	0.167	0.154
Self-rated L2 proficiency	0.053	0.041	0.032
Length of translation-learning experience	0.080	0.052	0.059
Weekly translation practice	0.047	0.033	0.039
Linear terms			
Persistence (*P*)		0.311***	0.297***
Confidence (*C*)		0.115	0.108
Higher-order terms			
*P* ^2^			–0.177*
*PC*			0.299*
*C* ^2^			–0.250**
*R* ^2^	0.017	0.160	0.183
Δ*R*^2^	0.017	0.143	0.023
*F*	0.99	8.71	7.96

Entries are unstandardized regression coefficients. Persistence and confidence were mean-centered before the polynomial terms were computed. Female was the reference category for gender, and translation-related major was the reference category for major type.

^*^p < 0.05, ^**^p < 0.01, and ^***^p < 0.001.

Model 3 further introduced the quadratic and interaction terms required for polynomial regression. This step increased the explained variance from 0.160 to 0.183, with an additional 2.3% of variance explained. In the full model, the linear effect of persistence remained positive and statistically significant (*b* = 0.297, *p* < 0.001), whereas the linear effect of confidence remained positive but non-significant (*b* = 0.108, *p*>0.05). More importantly, all three higher-order terms were statistically significant, including the quadratic term for persistence (*b* = −0.177, *p* < 0.05), the interaction term between persistence and confidence (*b* = 0.299, *p* < 0.05), and the quadratic term for confidence (*b* = −0.250, *p* < 0.01). Taken together, these results indicate that the joint relation of persistence and confidence to engagement was not adequately represented by a simple additive pattern, and that their configuration carried explanatory value beyond their linear effects alone.

To clarify the shape of this joint association, the polynomial regression coefficients were translated into the conventional response-surface parameters. As reported in Table 8, the slope along the line of congruence was positive and statistically significant (*a*_1_ = 0.405, *SE* = 0.047, *t* = 8.552, *p* < 0.001). This result indicates that engagement increased as persistence and confidence rose together. At the same time, the curvature along the congruence line was significantly negative (*a*_2_ = −0.129, *SE* = 0.047, *t* = −2.752, *andp* = 0.006), suggesting that the positive association along this line was not purely linear and tended to level off at higher levels of congruence.

**Table 8:** Response-surface parameters for engagement in translation learning.

Parameter	Interpretation	Estimate	SE	*t*	*p*
*a*_1_	Slope along the line of congruence (*P* = *C*)	0.405	0.047	8.552	< 0.001
*a*_2_	Curvature along the line of congruence (*P* = *C*)	–0.129	0.047	–2.752	0.006
*a*_3_	Slope along the line of incongruence (*P* = −*C*)	0.189	0.114	1.650	0.099
*a*_4_	Curvature along the line of incongruence (*P* = −*C*)	–0.727	0.238	–3.056	0.002

*P*, persistence; *C*, confidence. A significantly negative *a*_4_ indicates that engagement decreases as the discrepancy between persistence and confidence increases.

Turning to the line of incongruence, the slope was not statistically significant (*a*_3_ = 0.189, *SE* = 0.114, *t* = 1.650, *p* = 0.099), indicating that the data did not provide statistically reliable evidence that the direction of the discrepancy was associated with different levels of engagement. In other words, the pattern did not differ significantly depending on whether persistence exceeded confidence or confidence exceeded persistence. Because this parameter was close to the conventional significance threshold, we conducted an additional bootstrap robustness check. Using 5,000 bootstrap resamples, the bootstrap standard error for *a*_3_ was *SE*_boot_ = 0.112, and the bias-corrected and accelerated 95% confidence interval included zero, BCa 95% CI = [−0.029, 0.416]. This result was consistent with the original response-surface test and supported a cautious interpretation of the non-significant directional effect. However, the curvature along the line of incongruence was significantly negative (*a*_4_ = −0.727, *SE* = 0.238, *t* = −3.056, *p* = 0.002). This finding indicates that engagement declined as the discrepancy between persistence and confidence became larger. Overall, these results suggest that engagement in translation learning is associated not only with persistence as an individual resource, but also with the extent to which persistence and confidence are aligned within the learner.

We further examined multicollinearity in the polynomial regression model. Variance inflation factors were calculated for the linear, interaction, and quadratic terms as well as the control variables. The VIF values ranged from 1.12 to 4.67, and all values were below the commonly used threshold of 10. These results indicate that multicollinearity was not severe enough to compromise the interpretation of the polynomial regression coefficients.

### 4.5 Response-surface interpretation

**Figure 1:**
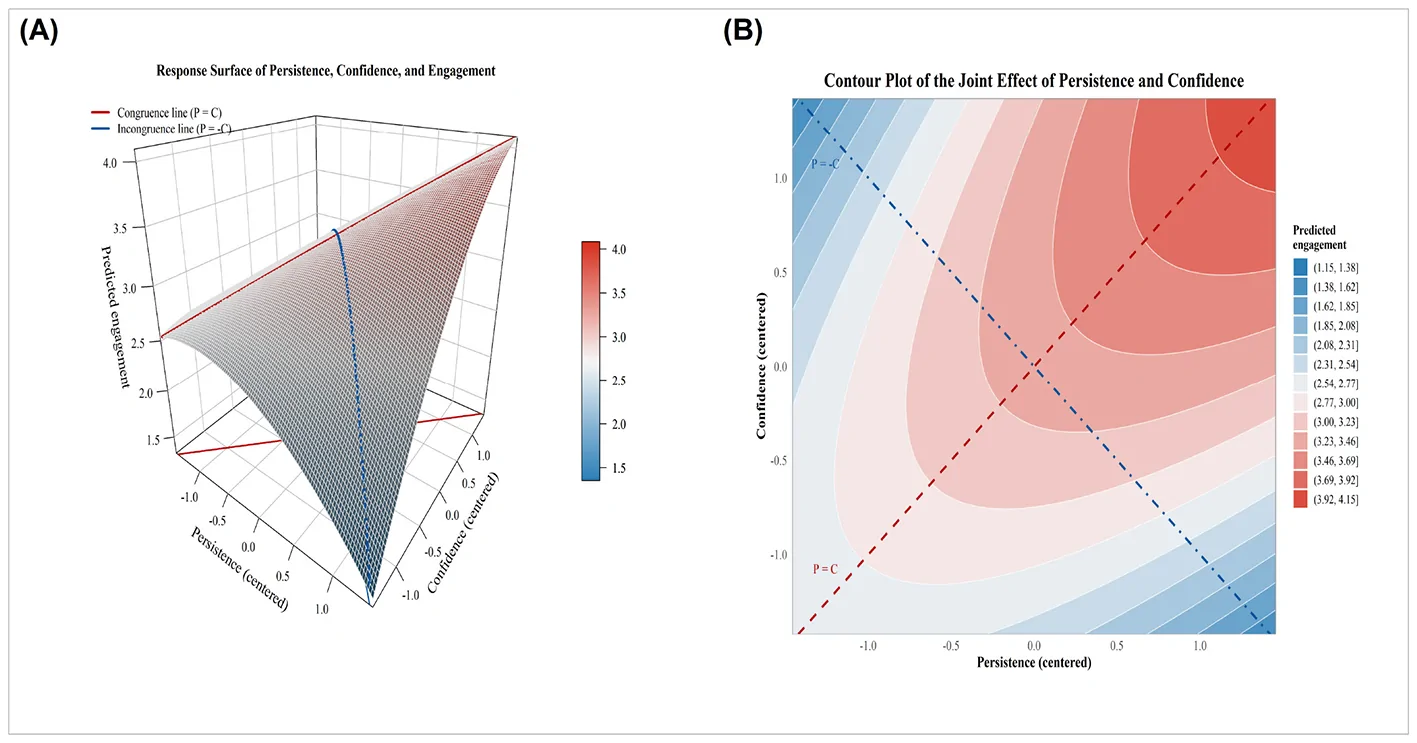
Response-surface results for the joint effect of persistence and confidence on engagement in translation learning. **(A)** presents the three-dimensional response surface. **(B)** presents the corresponding contour plot. The plots were generated from the full polynomial regression model (Model 3). For visualization, covariates were held constant at their sample means for continuous or ordinal variables and at their reference categories for categorical variables. The predicted values therefore represent model-implied engagement under these covariate settings rather than raw observed engagement. The red dashed line indicates the line of congruence (*P* = *C*), and the blue dash-dotted line indicates the line of incongruence (*P* = −*C*).

Figure 1 provides a visual representation of the joint effect of persistence and confidence on engagement in translation learning. As shown in Panel A, the response surface rises along the line of congruence (*P* = *C*), moving from the low-persistence and low-confidence corner toward the high-persistence and high-confidence corner. This pattern indicates that engagement becomes stronger as persistence and confidence increase together. At the same time, the surface does not rise in a perfectly straight fashion. Rather, the upward trend becomes less steep at higher levels of congruence, which is consistent with the significantly negative curvature along the congruence line reported in Table 8. In substantive terms, this suggests that the beneficial effect of jointly high persistence and confidence remains positive, but the incremental gain in engagement becomes smaller at the upper end of the surface. The downward bend along the line of incongruence (*P* = −*C*) is also clearly visible. As persistence and confidence move further apart, predicted engagement declines. This pattern is especially evident in Panel B, where the warmer region is concentrated around the area in which the two resources are relatively aligned and comparatively high, whereas cooler regions emerge as the discrepancy between them widens. This visual pattern accords with the significantly negative curvature along the incongruence line and provides direct graphical support for the central argument of the study that psychological mismatch carries a cost for engagement in translation learning. It is also noteworthy that the contour pattern does not show a strong directional imbalance on either side of the incongruence line. This visual impression is broadly consistent with the non-significant slope along that line. Although there is a slight tendency for the surface to tilt, the overall pattern suggests that the decline in engagement is driven mainly by the size of the discrepancy between persistence and confidence rather than by whether persistence exceeds confidence or confidence exceeds persistence. Taken together, the response-surface results reinforce the conclusion that engagement in translation learning is highest when persistence and confidence are aligned and jointly elevated, and that it weakens as the two psychological resources move out of balance.

## 5 Discussion

### 5.1 Overview of the main findings

This study examined whether engagement in translation learning is associated not only with persistence and confidence as separate psychological resources, but also with the degree to which these resources are aligned within the learner. Several findings deserve attention. First, persistence, confidence, and engagement were all positively associated at the zero-order level, suggesting that students who reported greater sustained effort and stronger capability beliefs also tended to report higher involvement in translation learning. Second, when the two resources were considered simultaneously, persistence emerged as the more robust linear correlate of engagement, whereas the linear contribution of confidence was weaker and did not reach conventional levels of statistical significance once persistence and the higher-order terms were taken into account. Third, the polynomial terms added a statistically detectable but modest increment in explained variance. Specifically, Model 3 increased *R*^2^ from 0.160 to 0.183, corresponding to an incremental Δ*R*^2^ of 0.023 beyond the linear model. This indicates that the configural component of the model provided additional explanatory information, but that the magnitude of this increment was modest relative to the larger contribution of the linear terms, particularly persistence. Fourth, response-surface analysis showed that engagement was generally higher when persistence and confidence were congruent and increased together. At the same time, the significant negative curvature along the congruence line suggested that the benefits of jointly high persistence and confidence were not unlimited, but gradually leveled off at the upper end of the surface. Finally, engagement was lower when the discrepancy between persistence and confidence was larger. This pattern supports a cautious conclusion that persistence–confidence incongruence is meaningfully associated with engagement, while also indicating that the practical magnitude of the configural effect should not be overstated.

These findings move the discussion beyond the familiar question of whether persistence or confidence matters more. Instead, they suggest that engagement in translation learning may be better understood by considering both individual psychological resources and their configuration. At the same time, the present findings do not imply that incongruence is the primary driver of engagement. Rather, the response-surface results provide an incremental and theoretically informative refinement to a model in which persistence remains the strongest linear correlate. This calibrated interpretation aligns with broader work in educational psychology that treats engagement as a situated form of behavioral, emotional, and cognitive involvement rather than as the simple by-product of a single motivational trait (Fredricks et al., 2004; Skinner et al., 2009; Kahu, 2013; Kahu and Nelson, 2018).

### 5.2 Theoretical interpretation of congruence between persistence and confidence

One of the clearest findings of the study was that engagement rose along the line of congruence. Students reported stronger engagement when persistence and confidence increased together, which suggests that effortful perseverance and capability beliefs can reinforce one another in translation learning. This result fits well with the view that engagement is sustained when students are both willing to continue and able to see themselves as capable of coping with academic demands (Bandura, 1993; Kahu and Nelson, 2018). In translation learning, such coordination is particularly important because tasks often involve ambiguity, iterative revision, delayed feedback, and the need to hold multiple linguistic alternatives in mind at once. Under these conditions, persistence without confidence may generate effort without stability, whereas confidence without persistence may support initial willingness but not sustained involvement. The present finding is also consistent with the emerging literature on persistence and confidence in language-related learning. Teimouri et al. (2022) showed that perseverance is a meaningful and domain-specific resource in second language learning, while research on translation self-efficacy has similarly suggested that learners' capability beliefs matter for how they approach translation tasks and how they interpret their progress (Yang et al., 2021; Haro-Soler and Kiraly, 2019; Lu et al., 2022; Abdel Latif and Alhamad, 2025). Our results extend this line of work by showing that the two resources should not be treated as wholly independent. What appears to matter most for engagement is not simply the presence of both, but their compatibility as a psychological system.

At the same time, the significant negative curvature along the congruence line adds nuance to this conclusion. Although engagement increased as persistence and confidence rose together, the slope became less steep at higher levels. This suggests a pattern of diminishing returns. In practical terms, moving from a low-low profile to a moderate or moderately high level of joint persistence and confidence may yield a meaningful gain in engagement, whereas further increases beyond that point may still be beneficial but produce smaller incremental changes. This pattern is psychologically plausible. Once students have reached a level at which they are both reasonably persistent and reasonably confident, additional gains in one or both resources may not translate into proportionally greater engagement because a substantial part of the motivational work has already been accomplished. In this sense, the present results suggest that engagement benefits not from maximal psychological intensification at all times, but from a sufficiently strong and coordinated motivational foundation.

### 5.3 Persistence–confidence incongruence and lower engagement in translation learning

A key finding of the study was the significant negative curvature along the line of incongruence. This result is best interpreted as a symmetric curvilinear association rather than as evidence of a causal cost. Students whose persistence and confidence were more discrepant at Wave 1 tended to report lower engagement 4 weeks later, after adjusting for the control variables. Because persistence and confidence were measured concurrently at Wave 1, the persistence–confidence configuration represents a baseline psychological profile rather than a mismatch process that unfolded over the course of the study. Thus, the two-wave design supports temporal ordering between the Wave 1 psychological configuration and Wave 2 engagement, but it does not establish that incongruence itself caused lower engagement. Substantively, this pattern suggests that translation learning engagement may be associated with a workable coordination between determination and perceived capability. Translation tasks often require students to sustain effort while dealing with ambiguity, delayed feedback, and repeated revision. When persistence and confidence are relatively balanced, students may be better positioned to maintain behavioral, emotional, and cognitive involvement. When the two resources are more discrepant, the motivational system may be less coherent, and students may find it more difficult to remain steadily engaged. This interpretation is consistent with the broader view that engagement is supported by coordinated motivational and self-regulatory processes rather than by isolated psychological resources alone (Bandura, 1993; Skinner et al., 2009; Kahu, 2013).

The present results do not show that one direction of incongruence is more consequential than the other. The slope along the line of incongruence was not statistically significant, indicating that the data did not distinguish between cases in which persistence exceeded confidence and cases in which confidence exceeded persistence. Therefore, these two directional profiles should be understood only as theoretically plausible examples of imbalance, not as empirically differentiated subgroups in the present study. The empirical support provided by the response-surface results concerns the magnitude of the discrepancy between persistence and confidence, as reflected in the significant negative curvature along the line of incongruence. This cautious interpretation also helps clarify the contribution of the study. Prior educational research using response surface analysis has shown that discrepancy itself can be associated with lower engagement in other learning contexts (Song et al., 2024). The present study extends this congruence-based logic to translation learning by showing that learner-internal incongruence between effortful persistence and capability-related confidence is associated with lower subsequent engagement. However, because the current study is observational, the finding should be understood as evidence of a prospective association rather than as evidence that psychological incongruence causally reduces engagement.

### 5.4 Why persistence was more robust than confidence

A *post-hoc* interpretation is needed to make sense of one noteworthy result: persistence showed a stable positive linear association with engagement, whereas confidence did not remain a significant independent linear predictor once persistence and the higher-order polynomial terms were included. This interpretation was not specified as a separate hypothesis in advance and should therefore be treated as an explanatory account of the observed pattern rather than as a confirmatory test. This pattern should not be interpreted as evidence that confidence is unimportant. Rather, it suggests that confidence may matter differently from persistence. In the present data, confidence seemed to contribute less as an isolated predictor and more as a resource whose educational value depends on how well it is aligned with persistence. This interpretation is compatible with general self-efficacy research. Self-efficacy beliefs are known to shape effort, resilience, and academic functioning (Bandura, 1993; Usher and Pajares, 2008; Honicke and Broadbent, 2016). Translation-specific studies also suggest that self-efficacy matters for both performance and the quality of students' learning processes (Yang et al., 2021; Lu et al., 2022; Haro-Soler and Kiraly, 2019; Abdel Latif and Alhamad, 2025). Yet the present findings indicate that, in translation learning, confidence may not always operate as a stand-alone motivational force strong enough to secure engagement by itself. This is understandable if one considers the structure of translation tasks. Students may believe they can handle translation problems in principle, but if they are not prepared to sustain effort through uncertainty, complexity, and revision, such confidence may not reliably translate into deep engagement. By contrast, persistence may provide a more direct basis for remaining behaviorally and cognitively involved even when confidence is less stable. In this sense, the present study offers a useful refinement rather than a contradiction of the self-efficacy literature. Confidence remains relevant, but its importance appears to lie partly in whether it is accompanied by sufficient persistence. This is precisely why the mismatch framework is informative. It shifts attention away from the question of whether confidence is globally important and toward the more psychologically precise question of when and under what internal conditions confidence is most likely to support engagement.

### 5.5 Practical implications for translation education

The present findings carry several implications for translation pedagogy. First, they suggest that educators should not treat engagement solely as a matter of instructional technique or task design. Although curricular structure and assessment practices clearly matter, engagement in translation learning also depends on how students' internal motivational resources are configured. Pedagogical efforts that focus exclusively on strategy training, text analysis, or technological competence may therefore miss an important part of the picture if they do not also attend to students' willingness to persist and their confidence in coping with translation-related demands.

Second, the findings suggest the value of identifying students whose persistence and confidence are out of balance. Some learners may work hard yet remain chronically doubtful of their capability, while others may express confidence but fail to sustain effort when tasks become demanding. These two profiles may look different on the surface, but both appear vulnerable in terms of engagement. Instructors may therefore benefit from paying attention not only to whether students seem motivated, but also to how their effort and confidence are combined. Classroom feedback, reflective journals, staged translation tasks, and structured peer interaction may all help teachers detect such mismatches more effectively (Haro-Soler and Kiraly, 2019; Yang et al., 2025; Wang, 2025).

Third, the results imply that support for translation learners should be psychologically calibrated. For students with relatively high persistence but lower confidence, feedback may need to emphasize attainable progress, mastery evidence, and concrete cues of growing capability. For students with relatively high confidence but weaker persistence, instruction may need to strengthen follow-through, revision habits, and tolerance for effortful problem solving. More broadly, the goal of teaching should not be to maximize any single psychological quality in isolation, but to cultivate a workable balance between determination and capability belief. Such an approach may be especially important in translation learning, where engagement is sustained over time through repeated cycles of drafting, monitoring, evaluating, and revising.

These practical implications should be interpreted cautiously. The present study does not provide a validated classroom diagnostic tool for identifying persistence–confidence mismatch at the individual-student level, nor does it establish cut-off scores that could be used to classify students into mismatch profiles. Therefore, the pedagogical suggestions offered here should be understood as general principles for teacher awareness and future intervention design rather than as ready-made diagnostic procedures. Future research should develop and validate brief screening, reflective, or feedback-based tools that can help instructors identify and support students whose effortful persistence and capability-related confidence appear to be out of balance.

### 5.6 Limitations and future directions

A first limitation concerns causal interpretation. Although persistence and confidence were assessed at Wave 1 and engagement was assessed 4 weeks later at Wave 2, persistence and confidence were measured concurrently at Wave 1. Accordingly, the persistence–confidence configuration should be understood as a baseline learner profile rather than as a temporally unfolding mismatch process. The design supports the temporal precedence of the Wave 1 predictors relative to subsequent engagement, but it does not establish that incongruence between persistence and confidence caused lower engagement. It remains possible that unmeasured antecedents, such as task difficulty, instructor support, prior performance feedback, prior translation performance, or classroom climate, contributed simultaneously to both the degree of alignment between persistence and confidence and later engagement. Future studies should use three-wave cross-lagged designs, repeated within-person measurement, experience-sampling methods, or pedagogical interventions to test whether changes in persistence–confidence alignment precede changes in engagement over time.

A second limitation concerns the generalizability of the findings. The present study was conducted with students from a single medical university in China, and the sample was characterized by a relatively high proportion of female students and a concentration of participants from English-related and translation-related majors. Although this sample was appropriate for examining persistence–confidence configurations within a translation-learning context, students from different institutional settings may differ in motivational profiles, career expectations, curricular structures, and learning experiences. Therefore, the present findings should not be assumed to generalize directly to students in comprehensive universities, dedicated translation and interpreting programs, or other educational contexts. Future research should replicate the present model using more diverse samples across institutions, disciplines, and cultural settings to examine the robustness and boundary conditions of the observed associations.

## 6 Conclusion

The present study examined whether engagement in translation learning is associated not only with isolated learner strengths, but also with the internal coordination of psychological resources. Among Chinese university students engaged in translation learning, persistence emerged as a stable positive correlate of engagement, whereas confidence appeared to matter less as an independent linear factor and more through its configuration with persistence. Engagement tended to be higher when persistence and confidence were aligned and jointly elevated, and it was lower when the discrepancy between the two resources was larger. These findings should be interpreted as evidence of a prospective association between a baseline persistence–confidence configuration and later engagement, rather than as evidence that psychological incongruence causes reductions in engagement. By applying a congruence perspective to translation learning engagement, this study extends current research beyond simple main-effect models and offers a more nuanced account of how learners remain involved in demanding translation tasks. The findings indicate that translation learning is not only a cognitive or pedagogical process, but also a psychological one, in which effort, self-belief, and internal balance are meaningfully associated with students' learning experience. From this perspective, supporting translation learners requires more than improving skills alone. It also requires attention to the psychological conditions under which persistence and confidence can work together in a coherent and productive way.

## Data Availability

The original contributions presented in the study are included in the article/supplementary material, further inquiries can be directed to the corresponding author/s.
